# The effect of pectin on swelling and permeability characteristics of free films containing Eudragit RL and/or RS as a coating formulation aimed for colonic drug delivery

**Published:** 2010

**Authors:** A. Akhgari, F. Farahmand, H. Afrasiabi Garekani, F. Sadeghi, T. Vandamme

**Affiliations:** 1 School of Pharmacy, Ahvaz Jundishapur University of Medical Sciences, Ahvaz; 2 School of Pharmacy, Mashhad University of Medical Sciences, Mashhad, Iran; 3 Institute Gilbert Laustriat, Département de Chimie Bioorganique, Faculté de Pharmacie, Université Louis Pasteur, Strasbourg, France

**Keywords:** Pectin, Eudragit, Colonic delivery, Permeability, Free film

## Abstract

**Background and the purpose of the study:**

The potential of pectin as a bacterially degradable polysaccharide for colon drug delivery has been demonstrated. Due to the high solubility and swelling properties of pectin in aqueous media, it is frequently used in combination with water insoluble polymers for targeting drugs to the colon. The aim of this study was to evaluate free films containing pectin as a bacterially-degradable polysaccharide in combination with Eudragit RL (ERL) and/or RS (ERS) as a coating formulation for colonic drug delivery.

**Methods:**

Isolated free films comprising 20% pectin and 80% ERL or ERS and their combination in 1:1 ratio were prepared by casting method. Then, free films were evaluated by water vapor transmission (WVT), swelling and permeability experiments for theophylline and indomethacin in different media.

**Results:**

Formulations containing ERL exhibited higher WVT, swelling and permeability compared with formulations containing ERS. The permeability of theophylline through free films composed of pectin and eudragit polymers in simulated colonic media was not significantly different from those obtained in other media. However indomethacin free films containing pectin and ERL showed higher permeation in simulated colonic fluid (SCF) compared to the other media.

**Major conclusion:**

Formulation containing pectin and ERL may be suitable as a coating formulation for colon targeted delivery of drugs of low solubility such as indomethacin.

## INTRODUCTION

One approach for colon specific drug delivery is utilization of materials which are degraded by the bacterial enzymes present in the large intestine. The potential of pectin as a bacterially degradable polysaccharide for colon drug delivery has been previously demonstrated (1–4). Pectin is a non-toxic water soluble gel-forming polysaccharide containing carboxylic acid groups. It is extracted from different sources, e.g. apple or citrus.

Combination of different polymers with unlike mechanism has been studied previously (5). Due to the high solubility and swelling properties of polysaccharides in aqueous media, their film coatings are unable to prevent the release of drugs from coated dosage forms during their transit through the stomach and the small intestine. Therefore a number of studies have been conducted to use coatings composed of polymethacrylates and polysaccharides such as inulin in order to prepare more suitable films for targeting drugs to the colon (6). Also, investigations on coatings containing cellulosic (Aquacoat ECD30, Surelease) or acrylic (Eudragit) insoluble polymer and pectin have been attempted (7–9). These combinations have advantages of their parent polymers and/or creation of useful properties. For example, the composite of pectin and ethyl cellulose combines the enzymatic susceptibility of pectin and the protective properties of ethyl cellulose. Composites of pectin and chitosan are enzymatically degradable and more water resistant; although the latter polymer is water soluble (10).

Combinations of pectin with ethyl cellulose, hydroxypropyl methylcellulose, and polycations such as ERL and ERS or chitosan (11) and also With drugs (12) have been reported previously. while combination of pectin and sustained release polymethacrylates have been used on coated pellets (13, 14), there are few studies on free films containing pectin and combination of ERL and/or ERS for colonic drug delivery (8). Studies on films or membranes are often used in screening tests to lower the number of formulations which are coated on dosage forms. Often, particles coated with a film behave differently from the film itself. However, there is no doubt that studies conducted on free films help to understand the behavior of dosage forms coated with these films (11). Moreover, analysis of some characteristics of polymers such as water vapor permeation and swelling which is essential in a drug delivery composite could be more precise by evaluation of polymeric free films.

The aim of this study was to prepare and evaluate characteristics of free films containing pectin as a bacterially-degradable polysaccharide in combination with time-dependent polymethacrylates ERL or ERS as a coating formulation for colonic drug delivery. Permeability experiments were carried out for two kinds of drugs; theophylline as a drug with relative high solubility and indomethacin as a drug model with low solubility. Also, permeability of water and swelling tests were carried out to evaluate free films.

## MATERIAL AND METHODS

### Material

Eudragit RS30D (ERS) and Eudragit RL30D (ERL) (Rohm Pharma, Germany), pectin (SKW Biosystems, France) with degree of esterification of 66%, pectinase (Pectinex Ultra SP-L) (Novozymes, Switzerland) and standard activity of 26000 PG/mL at pH 3.5, triethyl citrate (Merck, Germany), indomethacin (Darupakhsh, Iran), theophylline monohydrate (Cooper Rhone, France), magnesium nitrate hexahydrate (Mg(NO_3_ )_2_ .6H_2_ O) (Merck, Germany), and phosphorus pentoxide (Acros organics, USA) were supplied from indicated sources.

### Methods

#### Preparation of free films

A specific amount of aqueous dispersion of ERS, ERL or 1:1 ratio of two polymers was plasticized by a fixed amount of triethyl citrate (1:6 ratio related to total polymer content) and dispersion was stirred by a magnetic stirrer for 5 hrs. A solution of pectin in water 3% w/v was gently added with continuous stirring to the dispersion of Eudragits. The final suspension was poured into two teflon plates. Plates were stored in an oven of 50 °C for 24 hrs to complete removal of water and then transferred to a desiccator with 100% relative humidity (RH) and left for 24 hrs. Then, free films were removed from the surface of plates, the thickness of free films was measured and samples with mean thickness values in the range 150–170 µm were selected. Formulations consisted of pectin-ERS (20–80), pectin-ERL (20–80) and pectin-ERS-ERL (20–40–40). The ratio 1:4 for pectin:eudragit had the most suitable free films. Formulations with the higher amounts of pectin were not physicochemically stable

#### Water vapor transmission test (WVT)

Water vapor transmission (WVT) of films was determined gravimetrically at 25°C to find the moisture barrier properties of free films at room temperature and also their tightness and homogeneity. Free films with appropriate dimensions were sealed to WVT cups containing 10 ml of distilled water. The cups were accurately weighed and placed in a desiccator containing silica gel and appropriate amounts of calcium chloride to create a climate of low relative humidity (approximately 0%). Then, the cups were re-weighed at determined intervals (24, 48, 72, 96 and 120 hrs) and the profile of mass change versus time was plotted for each free film. WVT was calculated using following equation (15):

$$\text{WVT}=\frac{wx}{tAP_{0}({\text{RH}}_{1}-{\text{RH}}_{2})}$$

where *w/t* is the mass change (flux, g/h) resulting from slope of profile of the mass change versus time; *x*, the film thickness (mm); A, the area of the film surface exposed to the permeant (m^2^); P_0_ , the vapor pressure of pure water (kPa); (RH_1_ –RH_2_ ) the relative humidity gradient.

#### Swelling experiments

A sample of each free film (1cm^2^) was dried in an oven at 50 °C for 24 hrs. Then, dried film was accurately weighed (± 0.0001 g) and immersed in a flask of dissolution test containing 250 ml of different media at 37 °C. At specific intervals, the swollen sample was withdrawn from the medium and weighed (± 0.0001 g) after wiping the excess surface water by a filter paper. Duration of the tests was 3 hrs for all formulations. Swelling index, I_s_ (%), was calculated according to a reported method (16) as follows:

$${\text{I}}_{\text{s}}(\%)=\frac{{\text{W}}_{\text{s}}-{\text{W}}_{\text{d}}}{{\text{W}}_{\text{d}}}\times 100$$

where W_d_ is the weight of the dried polymer film and W_s_ denotes the weight after swelling. The media which were used for swelling experiments simulated gastric fluid (SGF) without pepsin, simulated intestinal fluid (SIF) without pancreatin with pH of 6.8 (USP XXVI) and simulated colonic fluid (SCF) by addition of 1 ml/l of pectinase to the media and of pH 6.4. All experiments were carried out in triplicate.

#### Permeability experiments

Isolated films of the polymers were mounted between the donor and acceptor compartments of a side-by-side diffusion cell (diffusion area 3.46 cm^2^) at 37 °C. For theophylline, the initial concentration of drug in the donor compartment was 3 g/l. The donor and acceptor compartments were both composed of SGF, SIF and SCF with pectinase. For indomethacin, SGF was not used as a medium because indomethacin was practically insoluble in acidic media. The initial concentration of indomethacin solutions in the donor compartment was 300 mg/l for SIF and SCF.

All permeability experiments were carried out for 3 hrs. At pre-determined time intervals, samples of 10 ml were taken from the receptor cells and replaced with fresh medium. The contents of the acceptor cells were assayed spectrophotometrically for theophylline and indomethacin at 272 and 318 nm, respectively. Each permeation experiment was carried out in triplicates and the cumulative amount of drug which was permeated following correction for the acceptor sample replacement was plotted against time.

Permeability of drug was defined according to the reported studies (17, 18) as:

$$\text{P}=\frac{{\text{d}}_{\text{M}}}{{\text{d}}_{\text{t}}\text{S}{\text{C}}_{\text{d}}}$$

Where M is the amount of the drug (mg) at time t; S, the effective diffusion area (cm^2^); C_d_ , the concentration of drug in the donor compartment and P is the permeability (cm/s).

#### Statistical analyses

One-way analysis of variance was used to assess the significance of the differences of the groups. Tukey- Kramer post test was used to compare the means of different treatment groups. Results with P<0.05 were considered statistically significant.

## RESULTS AND DISCUSSION

### 

#### Water vapor permeability tests

**Figure 1:**
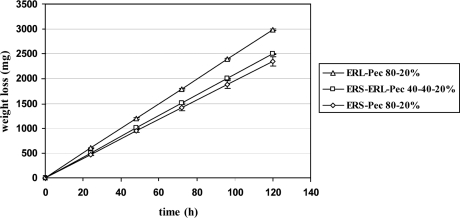
Profile of water vapor transmission through different free films of pectin and Eudragit^®^ RL-RS (Bars represent standard deviation).

**Figure 2:**
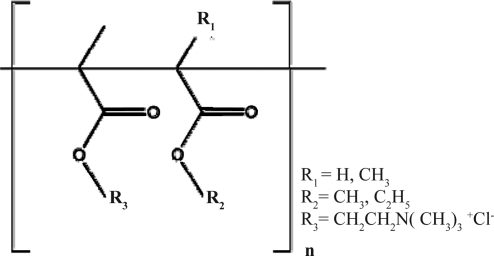
Eudragit RL and RS molecules.

Figure 1 depicts the profile of water vapor permeation for different free films. According to Fig. 1 water vapor transmission rate was constant for all free films. Table 1 illustrates the results of WVT experiments for all formulations. As depicted in Fig. 1 and Table 1, free films containing pectin and ERS showed more resistance to water vapor permeation than the other formulations. This is the result of less permeable character of ERS compared to ERL due to lower amount of quaternary ammonium groups in its structure. Addition of 40% ERL increased the rate of water vapor transmission significantly (p<0.01). According to Fig. 2, ERL has more quaternary ammonium groups in its structure compared to ERS and therefore, it is predictable that increase in the ratio of ERL in formulation could increase WVT. Formulations containing pectin and ERL had highest WVT values (p<0.001). Meanwhile, water vapor permeability of total formulations was relatively high and it was not much different for formulations. This could be due to the presence of carboxylic groups in pectin which makes it hydrophil. An increase in the hydrophilicity of free films could happen due to the larger number of hydroxyl groups available for interaction with molecules of water. The increase in WVT by addition of polysaccharide to polymeric films has been demonstrated in the other studies (19–21).

**Table 1:** vapor transmission of free films containing pectin-Eudragits.

Formulation	Film thickness (mean± SD; n=15) (µm)	Mass change (mg/h)	WVT (mean± SD; n=3) (mg.mm/m^2^ h kPa)
ERS- Pectin 80-20%	160±8	19.53	10.305±0.415
ERS-ERL-Pectin 40–40-20%	170±5	20.85	11.688±0.034
ERL-Pectin 80–20%	160±6	24.88	13.124±0.095

#### Swelling experiments

The results of swelling experiments are depicted in Table 2. As it is shown, free films containing pectin- ERS showed least swelling in different media due to the less permeability of ERS polymer to water. Addition of ERL to the formulations increased the swelling index significantly (p<0.001). The order of swelling ratio in different media for different Eudragits was as follows:

**Table 2:** Swelling index of free films in different media (data represent mean±SD; n=3).

Formulation	Maximum swelling index, I_s_(%)		

	SGF	SIF	SCF
ERS-Pectin 80–20%	56.1±3.5	59.3±2.5	54.8±154.8±1.2
ERS-ERL-Pectin 40-40-20%	84.1±2.9	74.2±0.7	7171.3±1.2
ERL-Pectin 80–20%	111.2±1.3	91.6±4.8	8 82.1±1.9

ERL>ERS-ERL>ERS

The amounts of swelling index for free films containing pectin-ERS in different dissolution media (SGF, SIF and SCF) were not significantly different from each other (p>0.05). This result was also observed for formulations composed of pectin-ERS-ERL. However for free films containing pectin-ERL, swelling ratio in the SCF was lower than that of SIF (p<0.01). Pectinolytic enzymes in simulated colonic media could degrade pectin in the film matrix and leaches it out of the film. This fact explains why the swelling index in SCF was lower compared to other media. This phenomenon could be more pronounced with free films containing ERL because this polymer is more permeable to water and therefore is able to extend its polymeric chains and expose pectin to the pectinase enzyme. Thus, free films containing ERL exhibited lower swelling ratio in SCF. Similar results have been reported by Semdé et al. (13). In their study on mixed pectin/ cellulosic or acrylic polymer coatings as carriers for colonic drug delivery, they observed that almost all the pectin in films consisting of pectin HM in combination with Aquacoat ECD30, Surelease, and Eudragit NE 30D released within 30 min into the media. However films of pectin and ERS 30D did not show this phenomenon.

#### Permeability experiments

Table 3 shows the results of theophylline permeability through different free films in various media. According to Table 3, free films containing ERS were less permeable to theophylline compared to those containing ERL and this phenomenon was found in all media (p<0.001). Formulations containing ERS-ERL had median permeability due to less permeability of ERS and therefore, there was higher resistance for movement of drug molecules across the film thickness. Permeability of theophylline through free films in different media (SGF, SIF and SCF) did not show any significant difference (p>0.05). The results indicated that free films containing pectin and ERS or ERL cannot be selective for permeation of water soluble theophylline in different media which is a disadvantage for dosage form intended to be used as a colonic drug delivery system.

**Table 3:** Permeability of theophylline through different free films (data represent mean±SD; n=3).

Formulation	P_theo_(x10^-5^cm/s)		

	SGF	SIF	SCF
ERS-Pec80-20%	3.757±0.085	3.591±0.158	3.734±0.118
ERS-ERL-Pec40-40-20%	4.212±0.065	3.923±0.169	4.250±0.152
ERL-Pec80-20%	4.464±0.105	4.223±0.258	4.549±0.061

The low permeability of free films consisting of pectin- ERL was in accordance with the data of water vapor permeation and could be due to some electrostatic interaction between pectin and Eudragit RL.

Table 4 shows the results of indomethacin permeability through different free films in various media. As depicted in Table 4, permeability of indomethacin in SIF was almost the same for all formulations (p>0.05). The low permeation of indomethacin from free films containing pectin- ERL despite of higher permeability of ERL could be attributed to the low water solubility of indomethacin which caused difficulty in drug penetration and movement through the polymeric chains. Therefore, high swelling of polymethacrylates could not effectively differentiate between the permeability of indomethacin through different free films effectively. In the SCF media, however free films containing ERL-pectin had higher permeation than ERS-pectin (p<0.01). Furthermore, permeability of these films in SCF was higher than those of SIF (p<0.05). This observation could be due to leaching of pectin out of the film in SCF. Pectinase enzyme which was presented in the SCF could break down the pectin from transdermal polymeric films could be due to leaching of polyvinyl pyrrolidone (PVP) from ethylcellulose/PVP films and formation of pores (22). Also, leaching of pectin from mixed films containing pectin, chitosan and HPMC has been demonstrated (23).

**Table 4:** Permeability of indomethacin through different free films (data represent mean±SD; n=3).

Formulation	P_indo._(x10^-5^cm/s)	

	SIF	SCF
ERS-Pec 80-20%	1.285±0.310	1.070±0.093
ERS-ERL-Pec 40-40-20%	1.188±0.056	1.445±0.278
ERL-Pec 80-20%	1.316±0.056	1.713±0.093

Films containing pectin-ERL were more permeable to indomethacin in SCF and therefore more sensitive to colonic medium than films of pectin- ERS. Another advantage for films composed of pectin-ERL was its lower permeation in the media which simulated upper GI tract (SGF and SIF) in spite of presence of higher permeable ERL in its composition. This is a property which is very useful for a colonic drug delivery system as it must be resistant to premature drug release in the upper parts of the GI tract.

## CONCLUSIONS

The results of this study revealed that addition of pectin to free films containing ERL modulated the high permeability of this polymethacrylate. Free films containing ERL in combination with pectin showed higher WVT and swelling data when compared with films containing ERS and pectin, however the difference was not as much as predicted which could be attributed to probable electrostatic interaction between pectin and ERL.

Permeability experiments for two model drugs demonstrated that the free films did not show significant differences in permeability of both drugs in SCF and the other media except for those films containing 20% pectin and 80% ERL which showed higher permeation in SCF for indomethacin. Based on this observation and also the resistance of film to the high swelling and permeation in simulated upper GI tract, this composition could be more suitable as a coating formulation for colon targeted delivery of drugs with low solubility such as indomethacin.
